# The clinical relevance of advanced artificial feedback in the control of a multi-functional myoelectric prosthesis

**DOI:** 10.1186/s12984-018-0371-1

**Published:** 2018-03-27

**Authors:** Marko Markovic, Meike A. Schweisfurth, Leonard F. Engels, Tashina Bentz, Daniela Wüstefeld, Dario Farina, Strahinja Dosen

**Affiliations:** 1 Department for Trauma Surgery, Orthopedics and Plastic Surgery, University Medical Center Göttingen, Von-Siebold-Str. 3, 37075 Göttingen, Germany; 2 Faculty of Life Sciences, University of Applied Sciences (HAW), Ulmenliet 20, 21033 Hamburg, Germany; 3 Biorobotics Institute, Scuola Superiore Sant’Anna, Viale R. Piaggio, 34, 56025 Pontedera (PI), Italy; 4 Georg-August University, 37075 Göttingen, Germany; 5 Otto Bock Competence Center, Otto Bock HealthCare GmbH, 37115 Duderstadt, Germany; 6 Department of Bioengineering, Imperial College London, SW7 2AZ, London, UK; 7 The Faculty of Medicine, Department of Health Science and Technology Center for Sensory-Motor Interaction, Aalborg University, Aalborg, Denmark

**Keywords:** Somatosensory feedback, Closed loop, Multi-functional, Upper limb prosthesis, Grasping, Amputee

## Abstract

**Background:**

To effectively replace the human hand, a prosthesis should seamlessly respond to user intentions but also convey sensory information back to the user. Restoration of sensory feedback is rated highly by the prosthesis users, and feedback is critical for grasping in able-bodied subjects. Nonetheless, the benefits of feedback in prosthetics are still debated. The lack of consensus is likely due to the complex nature of sensory feedback during prosthesis control, so that its effectiveness depends on multiple factors (e.g., task complexity, user learning).

**Methods:**

We evaluated the impact of these factors with a longitudinal assessment in six amputee subjects, using a clinical setup (socket, embedded control) and a range of tasks (box and blocks, block turn, clothespin and cups relocation). To provide feedback, we have proposed a novel vibrotactile stimulation scheme capable of transmitting multiple variables from a multifunction prosthesis. The subjects wore a bracelet with four by two uniformly placed vibro-tactors providing information on contact, prosthesis state (active function), and grasping force. The subjects also completed a questionnaire for the subjective evaluation of the feedback.

**Results:**

The tests demonstrated that feedback was beneficial only in the complex tasks (block turn, clothespin and cups relocation), and that the training had an important, task-dependent impact. In the clothespin relocation and block turn tasks, training allowed the subjects to establish successful feedforward control, and therefore, the feedback became redundant. In the cups relocation task, however, the subjects needed some training to learn how to properly exploit the feedback. The subjective evaluation of the feedback was consistently positive, regardless of the objective benefits. These results underline the multifaceted nature of closed-loop prosthesis control as, depending on the context, the same feedback interface can have different impact on performance. Finally, even if the closed-loop control does not improve the performance, it could be beneficial as it seems to improve the subjective experience.

**Conclusions:**

Therefore, in this study we demonstrate, for the first time, the relevance of an advanced, multi-variable feedback interface for dexterous, multi-functional prosthesis control in a clinically relevant setting.

**Electronic supplementary material:**

The online version of this article (10.1186/s12984-018-0371-1) contains supplementary material, which is available to authorized users.

## Background

The human hands are an essential and sophisticated instrument for stable grasping, dexterous manipulation, haptic exploration as well as social contact and communication. These functions are possible thanks to a rich network of feedforward (motor) and feedback (sensory) pathways connecting the brain and the hand. Acquired or congenital loss of the hand has a profound impact on the life of the affected. To restore the missing functions, patients are often equipped with myoelectric prostheses, which aim at replacing the human hand morphologically and functionally. Such prostheses are controlled by translating muscle signals, e.g., the contraction of wrist flexors and extensors, into closing and opening of the prosthetic hand. This allows intuitive control as the mapping between the muscles and the resulting movements is preserved as before the amputation. However, while state-of-the-art (SoA) commercial myoelectric prostheses do provide control of a dexterous, multi degrees of freedom (DoF) hand, thereby restoring the motor function, an effective method to provide sensory feedback is still missing [1–3].

Prosthesis users explicitly indicate the recovery of sensory capabilities through artificial sensory feedback as an important priority [4–9]. One survey reported that the users rated prostheses as functionally most unsatisfying in the tasks that required high dexterity, and they indicated the need for explicit feedback during those tasks [9, 10]. Moreover, since sensory input is so instrumental in normal human motor control [11, 12], it is generally assumed that meaningful feedback from hand prostheses would increase their utility [3, 13]. Yet, even after decades of research (the first feedback system was developed in the early 1950s [14]), no commercial implementation of artificial sensory feedback is available.

Over decades, researchers have explored several methods for providing feedback. The approaches can be invasive and non-invasive (recently reviewed by Svensson et al. [1]; here, we focus on the latter). To close the control loop, a prosthesis needs to be equipped with proprioceptive (joint angles) and exteroceptive (grasping force) sensors. The sensor data are read online and translated into stimulation profiles which are then delivered to the sensory motor structures available after the amputation. In a non-invasive approach, the stimulation is applied to the skin of the residual limb using electrical currents, vibration motors, and force and torque applicators. In most studies on sensory feedback, grasping force was the variable transmitted to the user. This is a reasonable choice, as the grasping force cannot be readily estimated in all cases using vision alone. As demonstrated before [15, 16], in routine grasping the resulting force can be estimated from the prosthesis closing velocity. However, this feedback cannot be used for force modulation once a rigid object has been grasped. In this case, the force increase is not followed by visually perceivable prosthesis movement.. The force magnitude was communicated to the user via parameter modulation, where the stimulation intensity and/or frequency was proportional to the measured force, spatial modulation, where each stimulator within a multichannel interface communicates a specific force range, or using mixed coding. As shown in [17], spatial and mixed coding occupy a larger area but allow easy interpretation of the feedback. In most studies, a single or at most two variables (grasping force and hand aperture) have been considered, and this is in marked contrast to contemporary prosthetic devices that are flexible systems with multiple functions (e.g., many grasps, active wrist or elbow function).

Feeding back sensor information to the user of the prosthesis is therefore relatively straightforward from the technological point of view. Nonetheless, the results in the literature on the benefits of feedback have been so far inconclusive and sometimes even contradictory. Only studies that fully blocked the vision and hearing (e.g., the subject wearing headphones and blindfold) consistently reported that feedback improved the performance whereas studies not blocking them showed inconsistent results. However, this is an expected outcome, as these studies compare the condition of full sensory deprivation to the condition in which the artificial stimulation was the only feedback source. Therefore, the performance with any feedback is likely to be better than with no feedback at all. When using a more realistic setup, on the other hand, there is no consensus regarding the benefits of feedback. Some researchers reported that non-invasive feedback was clearly beneficial [18–21] but sometimes the improvement was observed only in experienced users and under certain conditions [22]. For example, in [23] electrotactile feedback significantly improved the force control even in the presence of abundant visual cues (e.g., compliant object). Similarly, a longitudinal study [24] reported that amputee subjects were consistently better in performing a delicate grasping task across multiple sessions when vibrotactile feedback was provided. However, and in striking difference to the previous studies, in some cases [15, 25–28] feedback did not bring any improvement over vision, even when using a low-impedance device affording fine force control [26] or while performing a dual-task drawing the visual attention away from the prosthesis [29].

We have identified three main factors that have not been sufficiently considered in the current literature, but that we deem critical to determine the usefulness of feedback. Our hypotheses are based on the principles of human motor control, in which anticipation and learning play a significant role [30]. As demonstrated in [31] in experiments on able-bodied subjects, after an initial training the subjects successfully scaled the prosthesis grasping forces (economical grasping) even in the condition of full sensory deprivation. We believe that the same factors are operative in an amputee controlling a prosthesis. First, as demonstrated by deafferented subjects, simple repetitive tasks can be accomplished without feedback [32]. Therefore, we hypothesize that artificial feedback is objectively useful only in sufficiently complex tasks. Second, able-bodied subjects develop feedforward control through practice (internal models [33], sensorimotor memory [30]), and there is an indication that amputees rely on similar mechanisms [34, 35]. In fact, two recent studies [36, 37] investigated the mechanisms of motor adaptation in the context of myoelectric control with simulated prosthesis dynamics. Hence, we assume that prosthesis users learn to improve their internal model of feedforward control over time, using the explicit, supplemental feedback they receive, but also the incidental feedback of the prosthesis (e.g., motor noise, vibration through the socket, vision) to complete a given task [13, 15, 24, 38, 39]. We suggest that, consequently, the improvement of prosthesis control through explicit feedback would decrease with increasing feedforward proficiency [38]. And third, the subjective impression of any complex form of feedback depends on the users’ full understanding of it. Hence, we argue that artificial feedback with more than one feedback variable can be perceived as pleasant, useful, and easy to understand if it is introduced gradually. Accordingly, we designed a longitudinal explorative study with a realistic setup to address these factors and test our hypotheses.

In summary, this explorative study aims to shed light onto the role and potential benefit of feedback in real-life myoelectric prostheses applications. In it, we investigate how the role and the benefit of feedback depend on the task complexity, training and learning, and how subjective experience and understanding of the feedback changes with its complexity. To this goal we propose a novel vibrotactile feedback scheme (VFS) that can transmit multiple variables to support the control of a multifunction prosthesis. We use an array of vibrators and mixed coding (spatial and intensity modulation) to communicate contact, active function and the level of prosthesis grasping force using tactile patterns that are clear to perceive. We demonstrate that the presented system provides functional benefits that are, however, determined by the context in which the feedback is used. Additionally, to ensure clinical relevance of our study, we tested limb-deficient instead of able-bodied subjects and used a realistic clinical setup, including custom made sockets, commercial prosthesis and embedded myoelectric control. We systematically assessed objective and subjective performance indicators of a scalable VFS for multi-DoF hand prostheses. Finally, the protocol evaluated the subjects’ control performance longitudinally (across several sessions), with a battery of functional tasks, and in different conditions. The amount (complexity) of feedback was increased gradually following the increase in the task demands.

## Methods

### Subjects

Six subjects (36 ± 12 yrs.), including five subjects with an amputation (traumatic) and one subject with congenital limb absence participated in the study, all with little or no prior experience with multi-DoF myoelectric prostheses (see Table 1). The first four subjects were admitted for treatment (prosthetic fitting) at the Otto Bock Competence Center, Duderstadt, and recruited to simultaneously participate in this study. Subjects five and six were recruited externally for experimental purposes only and therefore were fitted with provisional sockets. Hence, the first four subjects were trained and treated by a professional therapist and sometimes (re)fitted with improved, refined versions of the prosthetics socket as their treatment progressed. Except for this difference, the two subject groups went through identical experimental sessions, supervised by the experimenter.

**Table 1:** Overview of subjects participating in the experiment

Subject	Age	Amputation level	Cause of amputation	Myoelectric prosthesis exp.	Multi-DoF prosthesis exp.
1^a^	37	Unilateral, transhumeral, non-dominant side	Traumatic, 2 years ago	None	None
2^a^	38	Unilateral, transhumeral, dominant side	Traumatic, 2 years ago	None	None
3^a^	25	Unilateral, transhumeral, dominant side	Traumatic, 2 years ago	None	None
4^a^	23	Unilateral, transhumeral, non-dominant side	Traumatic, 2 years ago	None	None
5	57	Unilateral, transradial, non-dominant side	Traumatic, 35 years ago	Active user, single-DoF	Little, < 30 h
6	25	Unilateral, transcarpal, non-dominant side	Congenital	Some, participated in experiments	Little, < 10 h

^a^Subjects that were simultaneously treated in the Otto Bock Competence Center, Duderstadt

### Experimental setup and feedback coding

**Fig. 1:**
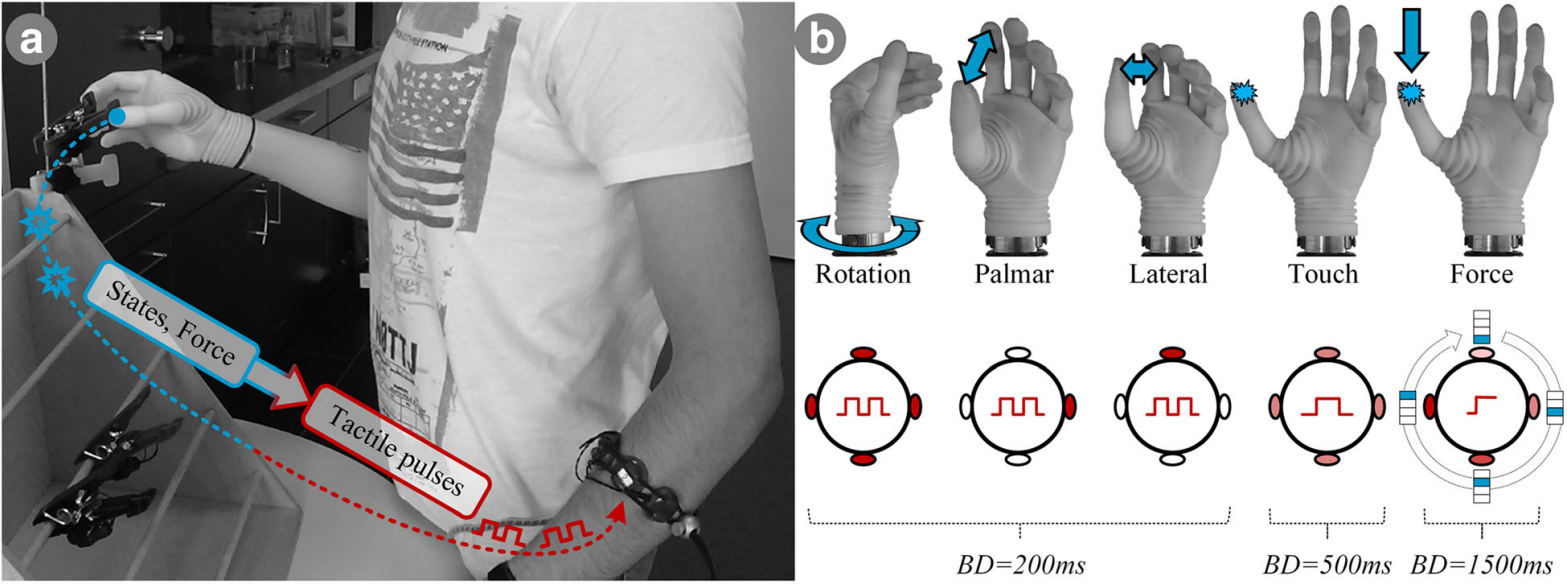
The placement (**a**) and coding (**b**) of the vibrotactile feedback coding scheme (VFS). The touch, DoF switching and grasping force (blue annotations) are coded into stimulation patterns (red annotations) presented on the contralateral side. The tactors can be active at a specified vibration intensity (light red – low intensity, dark red – high intensity) or inactive (white). Activation pattern (red pulses) can be either two short bursts, one burst, or a single prolonged burst. The information was coded using vibration bursts of different duration, location and amplitude (see text for explanation). Abbreviations: BD (burst duration) – the duration of a vibration burst

The experimental setup consisted of three components (Fig. 1a): 1) A Michelangelo hand prosthesis with a wrist rotator [40] and two 13E200 dry EMG electrodes with integrated amplifiers (Otto Bock Healthcare GmbH, Vienna, AT) [41], 2) eight C3 Tactors (Engineering Acoustics, Inc., Casselberry, Florida, USA) providing vibrotactile stimulation, integrated in an adjustable rubber band, and 3) a standard desktop PC with an Intel i5 processor running Windows 7 OS and MATLAB 2015b (MathWorks, Natick, US-MA). All experiments were performed in an upright position in front of a table that could be adjusted vertically to the height of the standing subject. The prosthesis socket covered the entire residual limb up to the shoulder in transhumeral subjects, and there was no space to place the vibration bracelet ipsilaterally. Therefore, it was positioned on the contralateral side, slightly below the elbow. As demonstrated in previous studies, it seems that human subjects can flexibly integrate feedback delivered to different body locations [20, 42–44].

The internal controller of the Michelangelo prosthesis provided commercial state-of-the-art two-channel sequential and proportional myoelectric control, with trigger-based (i.e., single- or two-channel bursts of EMG activity) switching between three available functions (DoFs): palmar grip, lateral grip, and wrist rotation. For four of the six subjects, the prosthesis was additionally equipped with a passive elbow, which was locked in 90° of flexion. The state machine operating the prosthesis, including the trigger type and the switching order between the DoFs, was configured for each subject individually, based on his/her preferences, and the configuration was not changed throughout the experiment. The prosthesis was instrumented with three position encoders (thumb, fingers, and wrist) and a single force transducer positioned at the base of the thumb, measuring the hand aperture, hand rotation and grasping force, respectively. The embedded prosthesis controller samples the sensor data and the processed EMG signals at the frequency of 100 Hz. The maximum prosthesis grip force was 70 N in palmar grip.

The eight C3 tactors were used to implement the vibrotactile feedback coding scheme (VFS). The host PC received the sensor data from the prosthesis and then translated this information in real time into the predefined patterns of stimulation (vibrotactile codes) delivered at four equidistant locations around the subject’s forearm (medial, lateral, ventral and dorsal side). This was achieved by simultaneously activating two tactors at each stimulation location (medial, lateral, ventral and dorsal tactor pairs). A C3 tactor has two control inputs that allow modulating the vibration amplitude and frequency of a tactor, which vibrates perpendicularly to the skin. Since the vibrations are generated using a mechanical oscillator including an electromagnet and a spring, the two parameters are not completely independent but coupled through a resonant effect. The maximum vibration displacement of the tactor is approximately 0.55 mm and the maximum frequency is 320 Hz. To transmit the full state of a multi DOF prosthesis, the VFS integrated multiple feedback variables of which some were discrete in nature (contact event, DOF switching) and some continuous (grasping force). The VFS was designed to be modular, allowing for arbitrary combinations of activation patterns. The feedback communicated the following information to the subjects: *‘Touch’* events, *‘DoF-switching’* events, and the prosthesis force (Fig. 1b). The vibration frequency was fixed at 180 Hz, which was a tradeoff between the optimal stimulation frequency of Pacinian corpuscles in the skin (250 Hz) [45] and the noise that the tactors produce while vibrating (a well-known drawback of voice coil vibration motors). The feedback information was transmitted using vibration bursts delivered at different locations and using different amplitudes (i.e., mixed spatial and amplitude coding), as explained below. The *‘Touch’* was detected when the prosthesis grasping force crossed the threshold of 3% of maximum force (rising edge), and this was indicated to the user by delivering a single 250-ms long vibration burst at 50% of the maximum amplitude at all four stimulation sites simultaneously. The importance of feedback on contact and release events for grasp control in able-bodied humans is well established [30] and it has been also demonstrated in prosthesis grasping in two recent studies [46, 47]. In the present study, however, the contact was coded differently (multiple vibrators, different burst duration) and the release has not been included (as the functional significance is unclear). The *‘DoF-Switch’* feedback comprised three discrete events, namely, switching into the lateral or palmar grasp and switching from grasping to wrist rotation control. These events were encoded by two short vibration bursts (two times 200 ms, with 100 ms of no vibration in between) at the maximal amplitude delivered through different tactor pairs, depending on the selected function: ventral and medial tactor pairs were activated to denote switching into lateral and palmar grasps, respectively, and the activation of all four pairs indicated the switch into rotation. The *‘Force’* feedback communicated five ranges of the grasping force. The first range was communicated by the aforementioned *‘Touch’* event. When the subject felt only the *‘Touch’* feedback, he/she knew that the grasping force was between 3 and 10%. The remaining ranges were represented using a combination of spatial and amplitude coding. To indicate that the grasping force was is in the ranges 11–24%, 25–39%, 40–59% and ≥ 60% of the maximum force, the tactor pairs were activated sequentially from ventral to medial side (ventral, lateral, dorsal and medial tactor pair, respectively) and the vibration amplitude was simultaneously increased (55%, 70%, 85% and 100%, respectively). The subjects could therefore rely on two cues to recognize the force range, the position of the activated tactor as well as the amplitude of vibrations. In order to prevent habituation [48], the force range was communicated as a single 1500-ms long vibration burst. The burst was delivered only if EMG activity was detected, indicating that the subject intended to operate the prosthesis, or if the force level had changed. It should be noted that if the generated grip force would rise abruptly above 10% then, consequently, the *‘Touch’* feedback would be circumvented and only the current force level would be communicated back to the user.

The host PC served as integration unit for data acquisition, processing, and recording. It ran MATLAB 2015b with a custom-built Simulink model that executed in hardware real time and used the custom-developed Closed-Loop Framework Toolbox [49] in order to acquire the data from the prosthesis and control the feedback. The prosthesis was controlled using a commercial real-time controller embedded into the socket. The prosthesis sensor data were transmitted to the PC wirelessly via the Otto Bock proprietary Bluetooth (BT) interface, while the commands to the C3 tactors were sent from the PC using a wired USB connection. The overall control loop operated at 100 Hz, with the BT communication delay of approximately 80 ms.

### Experimental tasks and outcome measures

**Fig. 2:**
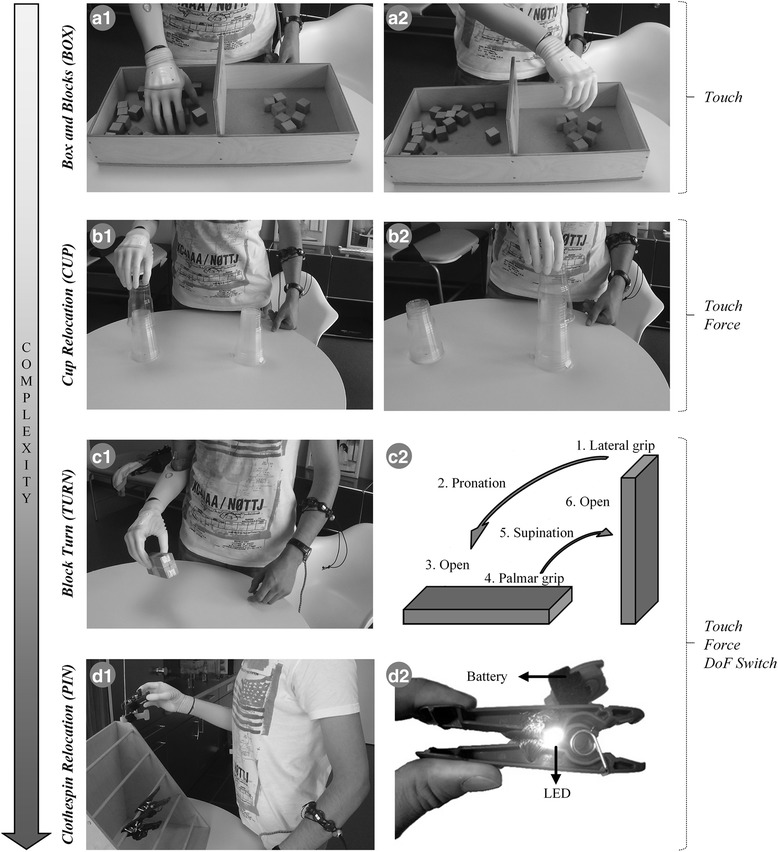
Overview of the experimental tasks and the corresponding feedback information communicated to the users (annotations on the right). The tasks are sorted in the order of increasing complexity, which was also how they were introduced to the subjects during the multisession experimental protocol (see section 2.4). (A) Standardized *Box and Blocks Task* (BOX), where the task was to transfer as many blocks as possible in 60 s from left (A1) to right (A2) compartment; (B) *Cups Relocation Task* (CUP) where the subjects needed to lift and pick up, one by one, 10 cups from the ipsilateral stack (B1) and transfer them, without dropping, to the contralateral stack (B2); (C) *Block Turn Task* (TURN) where the subjects needed to perform a set of dexterous actions (C2) manipulating the wooden block; and (D) *Clothespin Relocation Task* (PIN), containing “breakable” clothespins that lit up (D2) when the force exerted on them was excessive. The subjects’ task was to remove each of the four differently colored clothespins from the horizontal bar and place them on the vertical bar without “breaking” or dropping them (D1)

Four experimental tasks were used for evaluation of the subjects’ control performance. The *Box and Blocks Task* (BOX, see Fig. 2a) is a well-established method for testing the manual dexterity of human subjects [50]. The test setup consists of a wooden box (53.7 cm by 25.4 cm) with a partition in the middle. The subjects were instructed to transfer as many wooden blocks (2.5 × 2.5 × 2.5 cm^3^) as they could from one side of the partition to the other in 1 min, and this was counted as one run of the task. During the BOX, the subjects needed to control only opening and closing of the prosthesis in palmar grip, without activating other functions. If they accidentally generated a trigger and switched the function, they were instructed to switch back to the palmar grasp and continue the test. During this task, the subjects received the ‘*Touch’* feedback. In contrast to the other three tests, the time was not paused if the user dropped a block during the transfer. The number of successfully transferred blocks and the number of dropped blocks (called “retries” in the following for consistency) were used as primary and secondary outcome measures, respectively.

The *Cups Relocation Task* (CUP, see Fig. 2b) was inspired by the tasks that are commonly used for training the subject to use a new prosthesis in the Otto Bock Competence Center. It tests the ability of amputees to handle fragile objects, in this case plastic cups. A total of 11 plastic cups were stacked and placed bottom-up on the table in front of the subject. To prevent movements of the stack along the table surface, the bottom-most cup was glued to the table. The subjects were instructed to take one plastic cup after another from a location ipsilateral to the amputation side and restack them again at the marked point located 30 cm towards the contralateral side. During the CUP, the users received *‘Touch’* as well as *‘Force’* feedback. The CUP demanded fine control of grasping, because the subjects were required to lift a single cup from the stack, which was possible only by producing small enough forces (< 3 N). To successfully accomplish the task, the subjects were supposed to close the hand so that they felt the *‘Touch’* stimulation only, indicating that the grasping force was within the first force range. If the feedback indicated the second or a higher force range, the subject knew that the exerted force was too high. If a cup was dropped during manipulation (force too low) and/or the subjects grasped multiple cups at the same time (force too high), the trial was unsuccessful and the time was paused until the cup(s) was returned to its initial position (hereafter called “retry”). The total time needed to transfer 10 cups (equaling one run; the 11th cup was glued to the table) and the number of retries were primary and secondary outcome measures, respectively.

The *Block Turn Task* (TURN, see Fig. 2c) was first described in [51] to assess the dexterity of control in transradial amputees. In the present study, the test was adjusted for transhumeral amputees. The test evaluates the subjects’ ability to switch between DoFs while manipulating a solid object. One run of the task comprised the following sequence of actions: grasping an upright standing wooden block (size 3 × 5 × 15 cm^3^) using a lateral grip, pronating the wrist by 90°, releasing the block by opening the hand, grasping it anew using a palmar grip, supinating the wrist (90°) to bring the block back to its original position and placing it upright on the table. Since the task requires reliable grasping as well as switching through all available DoFs, the users were provided with the full feedback including *‘Touch’*, *‘DoF-Switch’*, and *‘Force’* information. If the block was dropped during manipulation or a wrong grasp was used, the trial was unsuccessful (“retry”) and the time was paused until the block was returned into the position corresponding to the end of the previous (successfully) completed action. The total time needed to finish the full sequence of actions (single run) and the number of retries during the run were used as primary and secondary outcome measures, respectively.

The *Clothespin Relocation Task* (PIN, see Fig. 2d) is an established method for evaluating the subjects’ ability to dexterously manipulate an object while maintaining a stable grasp. The task, first described in [52], includes relocating five plastic pins with different spring resistances from a horizontal to a vertical bar. The subjects therefore needed to take each pin using palmar grasp, supinate that arm (90°), release the pin by opening the prosthesis, and then pronate back to take the next pin. In principle, the subjects could grasp each of the pins using maximum force. To avoid this and require that the subjects control the grasping force, we equipped each pin with a switch and a small LED. If the subjects produced an excessive force during grasping, the pin opened too much, closing the switch and activating the LED (see Figure 2D2) [53]. An activated LED simulated the breaking of the clothespin and forced the subjects to repeat the grasp. Therefore, to successfully complete the relocation, the users needed to apply the right amount of force to open the clothespin enough so that it can be removed from the rack, while avoiding excessive force that would trigger the simulated breaking (see Table 2). Since this task required not only dexterous object manipulation but also fine regulation of force, the subjects received the full feedback (i.e., *‘Touch’*, *‘DoF-Switch’*, and *‘Force’*). If during transfer the clothespin was dropped or broken or a wrong grasp was used, the trial was unsuccessful (“retry”) and the time was paused until the clothespin was returned to its initial position. One run of the task comprised relocating four pins of different colors (i.e., spring stiffness and breaking force). The total time that the subjects needed to transfer all four clothespins and the number of retries within the run were taken as the primary and secondary outcome measures, respectively.

**Table 2:** Summary of minimal and maximal forces/apertures^a^ allowed for clothespins used in the PIN test

Pin color	Min aperture [%]	Max aperture [%]	Aperture window size [%]	Min force [%]	Max force [%]	Force window size [%]
Yellow	33	71	38	7	15	8
Red	33	66	33	13	23	10
Green	33	57	24	23	32	9
Black	33	57	24	35	43	8

^a^The values are relative to the prosthesis’ maximal grip force (70 N) and the clothespins’ maximal aperture (3.2 cm)

In summary, the two outcome measures characterizing each run in each task were 1) the run completion time in TURN, CUP and PIN or the number of transferred blocks in BOX, as the primary outcome measure, and 2) the number of retries per run, as the secondary outcome measure. Importantly, the primary and the secondary measures were not fully independent. Although the time was paused when the subjects made an error, the run completion time still included the time from the beginning of the run/action until the error had been made, e.g., from reaching to grasp a clothespin to dropping or “breaking” the object. Therefore, the primary measure was used to assess performance, whereas the secondary measure was employed as an additional check. Specifically, it was deemed that the performance improved only if a subject decreased the run completion time without simultaneously increasing the number of retries. On the contrary, a significant decrease in completion time with an increase in the number of retries was not considered as an improvement in performance.

As previously stated, the amount of feedback was adjusted to the demands of the experimental task. This was done to avoid providing feedback irrelevant for the task execution and, more importantly, to gradually introduce the subject to the tactile feedback.

### Experimental protocol

**Fig. 3:**
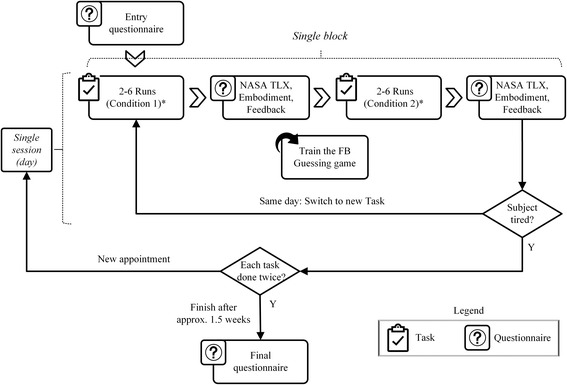
Overview of the experimental protocol. After filling in the entry questionnaire, the subjects started the experimental protocol that spanned several days (sessions), where each session included one or multiple experimental tasks performed with (FB) and without (NFB) supplemental feedback. After each condition, the subjects filled in the questionnaires. The protocol was finished once the subject performed each experimental task in at least two sessions. The last step in the study was to fill in the final questionnaire. (* denotes that the starting condition [FB/NFB] was randomized)

The total length of the experiment was around 1.5 weeks (Fig. 3). Upon admission to the study, the subjects were presented with an entry questionnaire (Additional file 1: Appendix I) that inquired about relevant personal data as well as the information on prosthesis-usage. After completing the entry questionnaire, the subjects performed a set of experimental tasks that were introduced in the order of increasing complexity (see Section 2.2): first Box and Blocks (BOX), then Cups Relocation (CUP) and Block Turn (TURN), and finally Clothespin Relocation (PIN). The experiment comprised several sessions (days) which comprised one or more blocks. Each block was dedicated to performing a single task, and the task was never repeated in a single experimental session. In each block, a single experimental task was performed in two conditions, with and without the supplemental feedback (FB and NFB). The tasks were scheduled so that each subject performed every task in at least two sessions. For example, in the first and second session, subject 2 performed one block of the BOX and in the third and fourth session, one block of the TURN. In the fifth session, subject 2 was tested in the PIN. In the sixth session, subject 2 performed one block of the TURN and the CUP, and in the seventh session one block of the CUP and the PIN. For comparison, subject 1 managed to perform one block of the TURN, CUP and PIN, all in a single (sixth) session. Such flexible organization of the experiment across days as well as within each session was necessary as the subjects had very different time constraints and abilities. In general, the subjects’ confidence increased over time and they could therefore perform more blocks per day. With the introduction of each new task, the subjects needed to control additional prosthesis functions (e.g., opening/closing in the BOX, opening/closing and wrist rotation in the TURN) and, to support this, the feedback provided more information (e.g., touch in the BOX, touch and active DoF in the TURN), as explained above (Fig. 1). Whenever new information was introduced in the feedback, the subjects received a brief training to recognize and interpret the novel coding. They played a 5-min long guessing game, which helped them to intuitively connect different stimulation patterns to specific prosthesis functions. The experimenter activated the feedback to deliver the information (e.g., a force level), and the subjects were asked to report verbally the meaning of the stimulation. After that, the experimenter would provide a correct answer (reinforced learning). This was done until the subjects could recognize the stimulation successfully several times in a row for each novel code. The novel feedback was trained using the guessing game only once, when a novel task in which that feedback was used was introduced for the first time.

The order of the conditions (FB, NFB) was randomized. Within each condition, a task was performed for a minimum of two and a maximum of six times (runs), depending on the subject’s individual performance and physical condition. The number of runs in each condition (FB, NFB) was identical. Once a condition was completed, the subject was presented with the extended, custom-modified NASA task-load index (TLX) questionnaire [54] (Additional file 2: Appendix II). In addition to the standard NASA-TLX questions, which assess the physical and mental task load on the subject, our modified version included questions regarding the benefit, comprehension, intelligibility, and sensation of the feedback (in FB condition only) as well as one question regarding the perceived prosthesis embodiment during the task execution. The experimental protocol was finished once a subject had performed each of the four experimental tasks in at least two sessions. If there was enough time and the subject was willing, the session was continued and additional blocks (with different tasks) were performed. Normally, the subjects would perform 2–3 blocks of each task. At the end of the experimental protocol, the subjects were presented with a final questionnaire that evaluated their overall impression about the feedback (Additional file 1: Appendix I).

The following outcome measures were used: 1) Two objective measures obtained in each run, reflecting the subject’s performance; 2) perceived workload, embodiment, and VFS performance (only in FB condition), measured subjectively once per condition in a respective session. Additionally, the subjects’ overall impressions about the VFS and feedback in general were evaluated in the final questionnaire that was presented to the subjects at the very end of the experimental protocol.

### Data analysis

The aim of the data analysis was to assess the influence of feedback on the task performance at the beginning (i.e., first session in which the task was performed) and the end of the experimental protocol (i.e., last session in which the task was performed). Depending on the subject, the last session was either the second or the third in the series (see Table 3).

**Table 3:** Summary of subject participation in the experimental protocol. All subjects except for the first two, participated in three sessions in each of the experimental tasks

Task	Total number of blocks / average number of runs per block and condition					
	Subject 1	Subject 2	Subject 3	Subject 4	Subject 5	Subject 6
Box and Blocks	2/2.5	2/3	3/4.5	3/2.5	3/3.5	3/3.5
Cups Relocation	2/3	2/3	3/6	3/2	3/3.5	3/3.5
Block Turn	3/4	3/3	3/6.5	3/3	3/3.5	3/3
Clothespin Relocation	3/4	2/3	3/3	3/2	3/3	3/3

For a given experimental task, the overall mean of the objective performance measures (completion time, retries) were calculated and presented for each subject individually by averaging the values of all runs that were performed within the same session (first and last) and condition (FB and NFB). The outcomes were then statistically compared between conditions separately for each task and session.

Since the questionnaires were presented once per condition, the subjective outcome measures were obtained by computing the average, subject-wise, across sessions and then pooling across tasks. The resulting data were statistically compared between the two conditions (FB and NFB). Finally, Questions 3–5 from the final questionnaire (Additional file 1: Appendix I), which assessed the subjective impression about the usefulness of *‘Touch’*, *‘Force’*, and *‘DoF-Switch’* feedback, respectively, were statistically compared to each other.

The data analysis was performed using MATLAB 2015b (MathWorks, Natick, US-MA) and since the data did not pass the normality test (Lilliefors test), the results are reported as median [inter-quartile range (IQR)]. The Wilcoxon signed-rank test was used for the comparisons between conditions (FB vs. NFB). A *p*-value of 0.05 was selected as the threshold for statistical significance. The scores for questions 3–5 from the final questionnaire were compared using the Friedman’s test.

## Results

In total, 67 blocks with an average of 3.5 ± 1.0 runs per block were recorded (Table 3). An equal number of data was collected for the two conditions (FB and NFB).

**Fig. 4:**
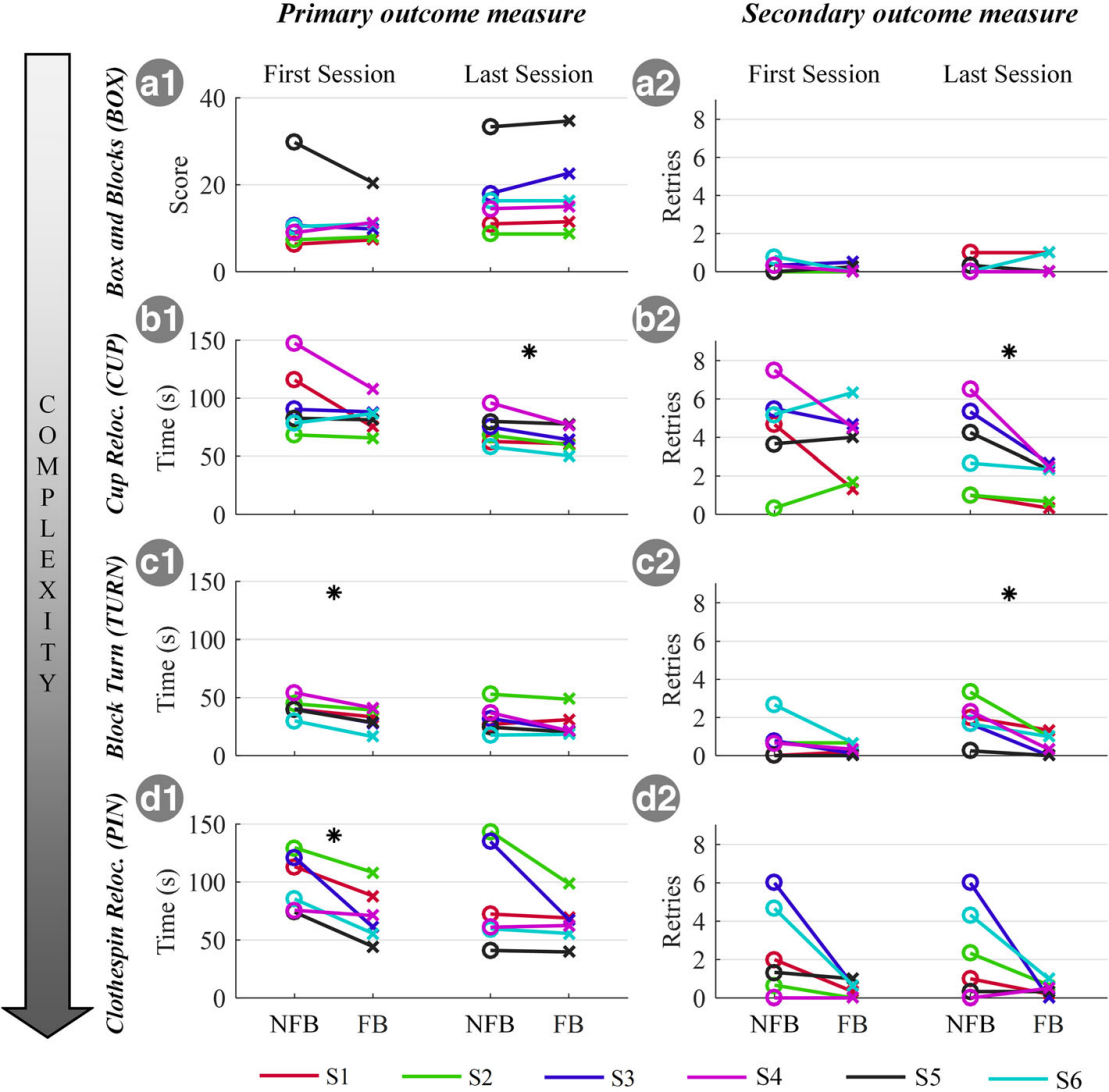
Individual subject results for the average run completion time/score (primary outcome) and the average number of retries per run (secondary outcome) across different experimental tasks, conditions (NFB/FB) and sessions. Please note that the conditions were not presented in the order shown here (NFB, FB) but randomized. Subjects are displayed in different colors. A star denotes the statistically significant differences (*, *p* < 0.05). Abbreviations: NFB – no supplemental feedback; FB – with supplemental feedback

The results for the objective performance measures are shown in Fig. 4, for each subject individually. The time to complete the task (primary measure) was significantly lower in the FB condition compared to NFB condition in the first session for Block Turn (TURN, 9 s faster, *p* = 0.031) and Clothespin Relocation Task (PIN, 33 s faster, p = 0.031) as well as in the last session for Cups Relocation Task (CUP, 9 s faster, p = 0.031). At the same time, in all these cases, the run completion time decreased without a significant increase in the number of retries, i.e., there was no significant difference in retries between FB and NFB and, in fact, the retries decreased in most cases. Therefore, when the feedback was provided, the subjects improved their performance in the first session of the TURN and PIN tasks and in the last session of the CUP task. In these cases, the subjects were faster in performing the task when the feedback was provided. The only task that did not show benefit from the supplemental feedback was the BOX.

**Fig. 5:**
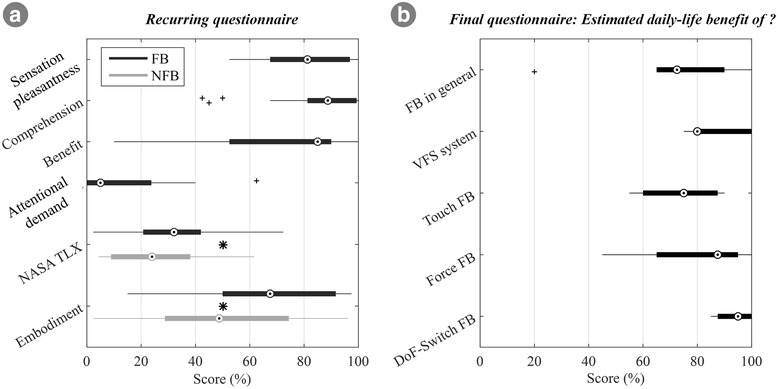
Summary of subjective ratings. The condition questionnaire (**a**) addressed the subjective impression of the feedback (sensation pleasantness, comprehension, benefit, and attentional demand, first four items) as well as the overall task workload (NASA TLX), and the prosthesis embodiment across different conditions (NFB/FB, last two items). The final questionnaire (**b**) summarizes the overall impression about the potential daily benefit of feedback in general, the present feedback coding scheme, and its different feedback variables (*‘Touch’*, *‘Force’*, *‘DoF-Switch’*). Boxplots depict the median (circle), interquartile range (box), maximal/minimal values (line) and outliers (+). Stars denote statistically significant differences (*, *p* < 0.05). Abbreviations: VFS – vibrotactile feedback coding scheme, NFB – without supplemental feedback; FB – with supplemental feedback

Figure 5a shows the overall subjective outcome measure, i.e., the questionnaire results, for both conditions. The subjects rated the feedback as comprehensible, pleasant, and beneficial, as reflected by the overall high median rating (> 80%) of the respective questions. Additionally, they rated the attention required to properly feel and interpret the feedback as rather low (median of ~ 5%). The additional attention demand, however, still contributed to the perceived overall workload that increased slightly but significantly from 24% in the NFB to 32% in the FB condition (*p* = 0.023). Finally, the subjects reported significantly higher scores for the overall feeling of prosthesis embodiment with feedback compared to no feedback (49% in NFB vs. 68% in FB, *p* < 0.001). In summary, the subjects perceived the feedback as comprehensible and useful. While it marginally increased the perceived workload, the overall sensation of prosthesis embodiment strongly increased. However, the results for some measures, especially perceived benefit and embodiment, exhibited a large dispersion and were subject- and task-specific.

The results from the final questionnaire (Additional file 1: Appendix I) are depicted in Fig. 5B. The subjects consistently expressed that the feedback in general (Q1 = 73%) as well as the specific coding scheme that they had tested in the present study (Q2 = 80%) would be beneficial in daily life. They valued all feedback variables (*‘Touch’*, *‘Force’*, *‘DoF-Switch’*) similarly (no significant difference) with an overall average median of ~ 85%.

## Discussion

To investigate the impact of task complexity and subject training on the role and the benefits of feedback, we have performed a longitudinal experiment using multi-functional prosthesis, advanced feedback scheme (VFS) and amputee subjects performing functionally relevant tasks. The evaluation addressed both objective (time or score, error rate) and subjective performance indicators at the beginning and end of task training (first and last experimental session with each task) and in two conditions (FB and NFB). Therefore, the study provides a comprehensive, multi-layered insight into different aspects of closed-loop prosthesis control with practical implications about the relevance of feedback across different tasks.

Our principal findings can be summarized in the following: 1) The benefit of the feedback depends on the task complexity. Only demanding tasks will profit from the feedback; the performance in simple tasks will remain unaffected as they can be accomplished by relying on incidental feedback sources (e.g., vision, sound). 2) The user experience has critical but complex impact on the usefulness of feedback. As the user becomes more acquainted with the task he/she might be able to improve his feedforward control to such an extent that the feedback becomes redundant. In some tasks, however, an opposite trend is possible, where only after the users learned how to properly utilize the myoelectric control, they were able to benefit from the feedback. 3) Users consistently experienced the feedback as useful, comfortable and pleasant and they considered that it would help them in their daily life. The present study therefore demonstrated the clinical advantages of the novel vibrotactile scheme designed for a multifunction prosthesis, revealing however that this impact is not consistent but depends on the context (training and task).

### Feedback interface

The developed vibrotactile feedback coding scheme (VFS) presents a novel and original approach for closing the loop in the context of a multi-DoF (3 DoFs) prosthesis control. To the best of our knowledge, this is the first feedback interface that was clinically evaluated in a series of functional tasks (BOX, CUP, TURN and PIN). Previous studies focused on the methods for feeding back a single variable, most often grasping force [3], and the assessment was typically conducted using simple prosthesis (a single DoF gripper). There are studies in which force and aperture were provided as feedback to control a single gripper and estimate stiffness. However, state of the art prosthetic systems integrate multiple functions. In a recent study [55], the electrotactile feedback was used to communicate flexion angles in the fingers of a dexterous robotic hand, but the assessment did not include functional tests with the hand mounted on the subject. The VFS in the present study introduces a novel feedback variable, e.g., an active state of the prosthesis to support DoF switching, and it is therefore suitable for use with a contemporary multi-DoF systems. The information transmission relies on the mixed coding which is easy to interpret, as demonstrated in our previous work [17]. Furthermore, the VFS provides 5-level force feedback which allows for fine object manipulation as demonstrated in the PIN and CUP tasks.

### The role and benefit of feedback

#### Feedback and task complexity

The results demonstrated that the benefit of feedback in the subjects naïve to the task (first session) depended on the type of task performed. The feedback improved performance in the first session of PIN and TURN but had no effect in BOX and CUP. The BOX is a simple task where the subjects controlled hand closing and opening. They could easily inspect visually if the prosthesis had grasped the object and, since the goal was to transport as many blocks as possible within the given time, they closed the prosthesis as fast as possible by strongly contracting the muscles. Since the prosthesis force is directly proportional to the muscle contraction, they generated firm, stable grasps easily, and the feedback information (*‘Touch’* event) was redundant (< 1 drop per run, Figure 4A2). Contrary to BOX, the TURN task required dexterity and skill in order to be executed fast and with minimal errors, as the subject needed to switch through the prosthesis DoFs several times in each run (3 DoFs, at least 5 switches per run). Without feedback (NFB condition in the first session), the subjects were unsure if they were in the correct DoF, and they were therefore cautious in handling the wooden block, thereby increasing the overall run time (Figure 4C1). As the feedback directly assisted the DoF switching, the subjects improved the performance. Finally, in the PIN task, the users had to perform less complicated switching between the DoFs (2 DoFs, at least 12 switches per run) but they needed to control the force, in order not to break the pin. Again, this resulted in a rather careful approach in the NFB condition of the first session, which increased the overall task execution time. Therefore, the benefit of feedback was expressed in the more complex tasks that required more challenging prosthesis control (PIN and TURN vs. BOX). In [31], the feedback did not provide any benefit in a simple task (grasping a light and heavy object), even when the subjects performed the task in the condition of full sensory deprivation.

#### Relevance of feedback as a function of subject’s skill

Since the feedback was provided in each session, it is possible that subjects used feedback to learn the task, and then used the acquired skills even when they didn’t have the feedback anymore. On the other hand, it is equally possible that they learned the tasks simply through repeated practice (with and without feedback). Due to the study design, it is not possible to decouple these two effects from the current results. Nevertheless, this study points out to some interesting conclusions concerning the relevance of feedback as a function of the overall user experience. On this discussion point, the way in which the subjects learned (i.e., training with feedback and/or repeated practice) is not relevant since the focus is on the relation between performance with feedback and prior subject skills. In CUP, the feedback became beneficial with training (last vs. first session), whereas the trend was opposite for TURN and PIN. In BOX, the training had no impact on the benefit of feedback. With training, the subjects could generate the small force levels (< 3 N) better, which was necessary to handle delicate objects (plastic cups) in CUP. Once they improved force control, they could also better exploit the feedback. This demonstrates that the feedback cannot be beneficial if the control is not sufficiently accurate. In a recent longitudinal study [24], the amputee subjects used a prosthesis equipped with contact feedback to perform a delicate grasping task, which is a similar context as in the present study with CUP. This previous study reported a similar trend as the present study, with the subjects improving the performance across sessions with feedback.

An opposite trend in the tasks PIN and TURN demonstrated that the feedback can become redundant with training. Initially, the subjects were naïve to the task as well as prosthesis control. The tasks were therefore challenging, and the additional information provided by the feedback was useful for control. With training, the subjects learned the task requirements and mastered the prosthesis control. For example, in TURN, they became confident in generating triggers and learned the order of the DoFs during DoF-switching. Furthermore, they might have learned how to exploit implicit feedback sources such as the distinct motor noise or vibrations through the socket, as well as visual cues, e.g., the clothespin aperture which was proportional to the applied force in PIN. All this provided the subjects with enough information, making the feedback redundant, and thereby, in the last session, they could complete the tasks with and without the feedback with similar performance. In a recent longitudinal study [38], the subjects trained to produce multiple levels of grasping force across sessions. The electrotactile feedback was beneficial, but the differences in the quality of open and closed-loop control (feedback vs. no feedback) decreased as the training progressed. Finally, the BOX is a task whose inherent characteristics -- simple control (open/close) and lack of constraints (robust blocks, no breaking) -- deemed the feedback redundant by default, and therefore the training did not bring any change in the utilization and benefit of feedback.

#### Subjective evaluation of feedback

Overall, subjects perceived feedback as useful, pleasant and easy to understand (Fig. 5a). Even though the additional attentional load of the VFS was rated as low, the overall task workload slightly increased in the FB condition. Importantly, this increase in workload had no negative effect on any of the objective performance measures. An interesting outcome is that the subjects consistently rated the prosthesis embodiment substantially greater in the FB than in the NFB condition. This occurred even if the feedback was not modality matched and despite being placed on the contralateral side due to technical constraints in this study. This observation may be due to the association between the feeling of prosthesis embodiment and perceived functionality. If the prosthesis did not react as the subject expected and intended, which happened more often in the NFB condition, the frustration level might increase, which might then negatively influence the feeling of embodiment. On the other hand, as embodiment was evaluated using a single question only (no control questions), the current results, even though they are in agreement with other recent evidence [56], remain to be confirmed in an additional study assessing detailed physiological data. This study could further shed light on the question whether integrating the feedback into the socket would further increase its usefulness and embodiment.

Future research should investigate the interaction between task learning and feedback and explore how each of the feedback variables (e.g., *‘Touch’*, *‘Force’*, *‘DoF-switch’*) contributes to the overall task performance. The subjective relevance of different feedback variables that were delivered simultaneously during prosthesis grasping was investigated in a recent study [53].

In summary, the present study demonstrates that the role and benefit of feedback is indeed a complex, multifaceted issue, which depends on the interaction between many factors. These need to be considered when investigating and designing effective feedback in prosthetics. During simple tasks, the users are not likely to profit from feedback since these are easily manageable by relying on implicit feedback sources and feedforward control. In complex tasks, in contrast, users can profit from supplemental feedback, but the extent might be limited by the users’ experience. The more experienced the users are in the task, the less they will need the additional feedback. However, in some tasks, users might benefit from the feedback only after a period of adaptation during which they improve prosthesis control and learn how to exploit the feedback. Interestingly, the experienced 1-DoF myoelectric user (Subject 5) and the congenital amputee (Subject 6) expressed similar trends across sessions and conditions as the naïve transhumeral amputees (Subjects 1–4) enrolled in the Otto Bock fitting program. In general, the subjective user impressions surpassed the objective benefits. The users rated positively almost any explicit feedback that they received, and they were motivated to learn even relatively complex feedback coding schemes (e.g., *‘DoF-Switch’*, *‘Force’*) if these were introduced gradually and appeared to be relevant for prosthesis operation. A similar result was reported in [25], where the users indicated that they experienced feedback as useful although there was no objective improvement in grasping performance. These results affirm that there is the need for shifting the development focus from simple, force-driven to complex, multi-functional feedback systems, which acknowledge that modern prostheses are dexterous devices.

## Conclusions

This study presents, for the first time, a non-invasive feedback coding scheme for multi-functional, dexterous prostheses. We measured objective and subjective benefits of the VFS in six upper-limb impaired subjects over several sessions. Objective benefit was measured with four functional tasks with increasing complexity, subjective benefit and load was measured with a modified NASA TLX after each task and a final questionnaire at the end of the study. The results showed that our multi-functional feedback was objectively useful in complex tasks, and subjectively useful in all tasks, regardless of the objective benefits.

Overall, the present study provides an important insight into the role and usefulness of feedback in the context of upper-limb prosthesis control. Moreover, it also presents an effective, multi-functional coding scheme on a small number of vibrotactile motors (four effectively), which makes it an ideal solution to be used in advanced dexterous prosthetic devices.

## Additional files

Additional file 1: Appendix I. Entry and Final questionnaire. (DOCX 19 kb)

Additional file 2: Appendix II. The extended NASA-TLX questionnaire. (DOCX 22 kb)

## Abbreviations

BD: Burst Duration
BOX: Box and Blocks Task
BT: Bluetooth
CUP: Cups Relocation Task
DoF: Degrees of Freedom
EMG: Electromyography
FB: Feedback
NFB: No Feedback
PIN: Clothespin Relocation Task
SoA: State-of-the-Art
TLX: NASA Task Load Index
TURN: Block Turn Task
VFS: Vibrotactile Feedback Scheme
