# Supplementary material for: The clinical relevance of advanced artificial feedback in the control of a multi-functional myoelectric prosthesis
Source: J Neuroeng Rehabil. 2018 Mar 27;15:28. doi: 10.1186/s12984-018-0371-1 (PMC5870217; doi:10.1186/s12984-018-0371-1)
Supplement: Supplementary file 1 — Appendix I. Entry and Final questionnaire. (DOCX 19 kb) [file 12984_2018_371_MOESM1_ESM.docx]

***Initial questionnaire*** Date:

| Name | Date of birth |  |
| --- | --- | --- |
|  |  |  |
|  |  |  |

| Was the amputated hand your dominant hand? | | 🞏 yes | 🞏 no |
| --- | --- | --- | --- |
|  |  | 🞏 ambidextrous | 🞏 no dominant hand |
| When was your amputation? | | ______________________  (month, year) | |
| Did you wear another prosthesis previously? | | 🞏 Cosmetic | If yes, for how long? _______ |
|  |  | 🞏 Body-powered | If yes, for how long? _______ |
|  | | 🞏 Myoelectric | If yes, for how long? _______ |
|  |  | 🞏 No |  |
| If yes: | How often did you wear your previous prosthesis? (please estimate both) | In a regular week (number of days): _______ | |
|  | | On a regular day (number of hours): _______ | |

The following two questions relate to your **previous prosthesis**:

|  | | | | How much did you perceive the previous prosthesis as your own hand? | | | | | | | | | | | | | | | | | | | | | | | | |
| --- | --- | --- | --- | --- | --- | --- | --- | --- | --- | --- | --- | --- | --- | --- | --- | --- | --- | --- | --- | --- | --- | --- | --- | --- | --- | --- | --- | --- |
|  |  |  | |  |  |  |  |  |  |  |  |  |  |  |  |  |  |  |  |  |  |  |  |  |  |  |  |  |
|  |  |  | | 🞏 |  |  |  |  |  |  |  |  |  |  |  |  |  |  |  |  |  |  |  |  |  |  |  |  |
|  |  | Not at all | | | | | Very little | | | | | | | | | | Very much | | | | | | | | | |  |  |
|  |  |  | |  |  |  |  | | | | | | | | | |  | | | | | | | | | |  |  |

|  | | | | Did you have the feeling that you get feedback from your previous prosthesis (for example about the grip strength) through vibration (through the socket), sound, or otherwise? | | | | | | | | | | | | | | | | | | | | | | | | |
| --- | --- | --- | --- | --- | --- | --- | --- | --- | --- | --- | --- | --- | --- | --- | --- | --- | --- | --- | --- | --- | --- | --- | --- | --- | --- | --- | --- | --- |
|  |  |  | |  |  |  |  |  |  |  |  |  |  |  |  |  |  |  |  |  |  |  |  |  |  |  |  |  |
|  |  |  | | 🞏 |  |  |  |  |  |  |  |  |  |  |  |  |  |  |  |  |  |  |  |  |  |  |  |  |
|  |  | Not at all | | | | | Very little | | | | | | | | | | Very much | | | | | | | | | |  |  |
|  |  |  | |  |  |  |  | | | | | | | | | |  | | | | | | | | | |  |  |

| Do you feel pain **at the site of the amputation** in this moment? | | | | | | | | | | | | | | | | | | | | | | | | | |
| --- | --- | --- | --- | --- | --- | --- | --- | --- | --- | --- | --- | --- | --- | --- | --- | --- | --- | --- | --- | --- | --- | --- | --- | --- | --- |
|  |  |  |  |  |  |  |  |  |  |  |  |  |  |  |  |  |  |  |  |  |  |  |  |  |  |
|  | 🞏 |  |  |  |  |  |  |  |  |  |  |  |  |  |  |  |  |  |  |  |  |  |  |  |  |
| Not at all | | | | Very little | | | | | | | | | | Very much | | | | | | | | | |  |  |

***Final questionnaire***

| Name | Date |  |
| --- | --- | --- |
|  |  |  |
|  |  |  |

| Do you think you could control your current prosthesis better if you received (more) feedback? | | | | | | | | | | | | | | | | | | | | | | | | | |
| --- | --- | --- | --- | --- | --- | --- | --- | --- | --- | --- | --- | --- | --- | --- | --- | --- | --- | --- | --- | --- | --- | --- | --- | --- | --- |
|  |  |  |  |  |  |  |  |  |  |  |  |  |  |  |  |  |  |  |  |  |  |  |  |  |  |
|  | 🞏 |  |  |  |  |  |  |  |  |  |  |  |  |  |  |  |  |  |  |  |  |  |  |  |  |
| Not at all | | | | Very little | | | | | | | | | | Very much | | | | | | | | | |  |  |
|  |  |  |  |  | | | | | | | | | |  | | | | | | | | | |  |  |

| Do you think you would profit from the additional feedback, which we provided, **in** **your everyday life**? |  |
| --- | --- |

|  |  |  |  |  |  |  |  |  |  |  |  |  |  |  |  |  |  |  |  |  |  |  |  |
| --- | --- | --- | --- | --- | --- | --- | --- | --- | --- | --- | --- | --- | --- | --- | --- | --- | --- | --- | --- | --- | --- | --- | --- |
|  | 🞏 |  |  |  |  |  |  |  |  |  |  |  |  |  |  |  |  |  |  |  |  |  |  |
| Not at all | | | | Very little | | | | | | | | | | Very much | | | | | | | | | |
|  |  |  |  |  | | | | | | | | | |  | | | | | | | | | |

| Can you think of any improvements, anything you would change in the feedback? |
| --- |

________________________________________________________________________________________________________________________________________________________________________________________________________________________________________________________________________________________________________________________________________________________________________________________________________________________________________________________________________________________________________________________________________________________________________________________________________________________________________________________________________________________________________________________________________________________________________________________________
