# Supplementary material for: The clinical relevance of advanced artificial feedback in the control of a multi-functional myoelectric prosthesis
Source: J Neuroeng Rehabil. 2018 Mar 27;15:28. doi: 10.1186/s12984-018-0371-1 (PMC5870217; doi:10.1186/s12984-018-0371-1)
Supplement: Supplementary file 2 — Appendix II. The extended NASA-TLX questionnaire. (DOCX 22 kb) [file 12984_2018_371_MOESM2_ESM.docx]

| **Name** | **Task** | **Date** |
| --- | --- | --- |
|  |  |  |

| 1. Mental demand | How mentally demanding was the task? |
| --- | --- |

|  |  |  |  |  |  |  |  |  |  |  |  |  |  |  |  |  |  |  |  |  |  |  |  |  |  |
| --- | --- | --- | --- | --- | --- | --- | --- | --- | --- | --- | --- | --- | --- | --- | --- | --- | --- | --- | --- | --- | --- | --- | --- | --- | --- |
|  |  |  |  |  |  |  |  |  |  |  |  |  |  |  |  |  |  |  |  |  |  |  |  |  |  |
|  |  |  |  |  |  | Very low | | | | | | | | | | Very high | | | | | | | | | |

| 1. Physical demand | How physically demanding was the task? |
| --- | --- |

|  |  |  |  |  |  |  |  |  |  |  |  |  |  |  |  |  |  |  |  |  |  |  |  |  |  |
| --- | --- | --- | --- | --- | --- | --- | --- | --- | --- | --- | --- | --- | --- | --- | --- | --- | --- | --- | --- | --- | --- | --- | --- | --- | --- |
|  |  |  |  |  |  |  |  |  |  |  |  |  |  |  |  |  |  |  |  |  |  |  |  |  |  |
|  |  |  |  |  |  | Very low | | | | | | | | | | Very high | | | | | | | | | |

| 1. Temporal demand | How rushed did you feel? |
| --- | --- |

|  |  |  |  |  |  |  |  |  |  |  |  |  |  |  |  |  |  |  |  |  |  |  |  |  |  |
| --- | --- | --- | --- | --- | --- | --- | --- | --- | --- | --- | --- | --- | --- | --- | --- | --- | --- | --- | --- | --- | --- | --- | --- | --- | --- |
|  |  | 🞏 | | |  |  |  |  |  |  |  |  |  |  |  |  |  |  |  |  |  |  |  |  |  |
|  |  | Not at all | | | | Very low | | | | | | | | | | Very high | | | | | | | | | |

| 1. Performance | How successful were you in accomplishing what you were asked to do? |
| --- | --- |

|  |  |  |  |  |  |  |  |  |  |  |  |  |  |  |  |  |  |  |  |  |  |  |  |  |  |
| --- | --- | --- | --- | --- | --- | --- | --- | --- | --- | --- | --- | --- | --- | --- | --- | --- | --- | --- | --- | --- | --- | --- | --- | --- | --- |
|  |  | 🞏 | | |  |  |  |  |  |  |  |  |  |  |  |  |  |  |  |  |  |  |  |  |  |
|  |  | Not at all | | | | Very low | | | | | | | | | | Very high | | | | | | | | | |

| 1. Effort | How hard did you have to work to accomplish this level of performance? |
| --- | --- |

|  |  |  |  |  |  |  |  |  |  |  |  |  |  |  |  |  |  |  |  |  |  |  |  |  |  |
| --- | --- | --- | --- | --- | --- | --- | --- | --- | --- | --- | --- | --- | --- | --- | --- | --- | --- | --- | --- | --- | --- | --- | --- | --- | --- |
|  |  |  |  |  |  |  |  |  |  |  |  |  |  |  |  |  |  |  |  |  |  |  |  |  |  |
|  |  |  |  |  |  | Very low | | | | | | | | | | Very high | | | | | | | | | |

| 1. Frustration | How insecure, discouraged, irritated, and/ or annoyed did you feel? |
| --- | --- |

|  |  |  |  |  |  |  |  |  |  |  |  |  |  |  |  |  |  |  |  |  |  |  |  |  |  |
| --- | --- | --- | --- | --- | --- | --- | --- | --- | --- | --- | --- | --- | --- | --- | --- | --- | --- | --- | --- | --- | --- | --- | --- | --- | --- |
|  |  | 🞏 | | |  |  |  |  |  |  |  |  |  |  |  |  |  |  |  |  |  |  |  |  |  |
|  |  | Not at all | | | | Very low | | | | | | | | | | Very high | | | | | | | | | |

| 1. Embodiment | Did you have the feeling that the prosthesis was part of your body? |
| --- | --- |

|  |  |  |  |  |  |  |  |  |  |  |  |  |  |  |  |  |  |  |  |  |  |  |  |  |  |
| --- | --- | --- | --- | --- | --- | --- | --- | --- | --- | --- | --- | --- | --- | --- | --- | --- | --- | --- | --- | --- | --- | --- | --- | --- | --- |
|  |  | 🞏 | | |  |  |  |  |  |  |  |  |  |  |  |  |  |  |  |  |  |  |  |  |  |
|  |  | Not at all | | | | Very low | | | | | | | | | | Very high | | | | | | | | | |

**If you did not receive feedback the questionnaire ends here.**

| 1. Benefit from the feedback | To what extent could you benefit from the feedback? |
| --- | --- |

|  |  |  |  |  |  |  |  |  |  |  |  |  |  |  |  |  |  |  |  |  |  |  |  |  |  |
| --- | --- | --- | --- | --- | --- | --- | --- | --- | --- | --- | --- | --- | --- | --- | --- | --- | --- | --- | --- | --- | --- | --- | --- | --- | --- |
|  |  | 🞏 | | |  |  |  |  |  |  |  |  |  |  |  |  |  |  |  |  |  |  |  |  |  |
|  |  | Not at all | | | | Very low | | | | | | | | | | Very high | | | | | | | | | |

| 1. Comprehension of the feedback | How well or poorly did you understand the feedback? |
| --- | --- |

|  |  |  |  |  |  |  |  |  |  |  |  |  |  |  |  |  |  |  |  |  |  |  |  |  |  |
| --- | --- | --- | --- | --- | --- | --- | --- | --- | --- | --- | --- | --- | --- | --- | --- | --- | --- | --- | --- | --- | --- | --- | --- | --- | --- |
|  |  | 🞏 | | |  |  |  |  |  |  |  |  |  |  |  |  |  |  |  |  |  |  |  |  |  |
|  |  | Not at all | | | | Very low | | | | | | | | | | Very high | | | | | | | | | |

| 1. Intelligibility of the feedback | How hard was it to focus on the feedback and to use it correspondingly? |
| --- | --- |

|  |  |  |  |  |  |  |  |  |  |  |  |  |  |  |  |  |  |  |  |  |  |  |  |  |  |
| --- | --- | --- | --- | --- | --- | --- | --- | --- | --- | --- | --- | --- | --- | --- | --- | --- | --- | --- | --- | --- | --- | --- | --- | --- | --- |
|  |  | 🞏 | | |  |  |  |  |  |  |  |  |  |  |  |  |  |  |  |  |  |  |  |  |  |
|  |  | Not at all | | | | Very low | | | | | | | | | | Very high | | | | | | | | | |

| 1. Sensation | How would you rate the sensation of the feedback? |
| --- | --- |

|  |  |  |  |  |  |  |  |  |  |  |  |  |  |  |  |  |  |  |  |  |  |  |  |  |  |
| --- | --- | --- | --- | --- | --- | --- | --- | --- | --- | --- | --- | --- | --- | --- | --- | --- | --- | --- | --- | --- | --- | --- | --- | --- | --- |
|  |  |  |  |  |  |  |  |  |  |  |  |  |  |  |  |  |  |  |  |  |  |  |  |  |  |
|  |  |  |  |  |  | Very unpleasant | | | | | | | | | | Very pleasant | | | | | | | | | |
